# Expert consensus on disease-based long-term follow-up care plans for childhood cancer survivors

**DOI:** 10.1007/s12519-025-00989-1

**Published:** 2025-10-30

**Authors:** Jiao-Yang Cai, Ching-Hon Pui, Xiu-Li Ju, Winnie Tso, Ya-Li Han, Wen-Ting Hu, Anthony Liu, Melissa M. Hudson, Yin Ting Cheung, China National Children’s Medical Center-Shanghai Long-Term Follow-Up (NCMCs-LTFU) Study Group, Jiao-Yang Cai, Xiu-Li Ju, Winnie Tso, Yin Ting Cheung, Frankie Cheng, Xin-Wei Zhu, Xiao-Wen Zhai, Yan Dai, Ai-Guo Liu, Ai Zhang, Xiao-Yan Wu, Fen Zhou, Hui-Rong Mai, Wei-Na Zhang, Jin-Qi Liu, Liu Qing, Hui Jiang, Jia-Shi Zhu, Yu Du, Li Hao, Shao-Yan Hu, Jia-Jia Zheng, Shao-Hua Le, Cai Chen, Xian-Min Guan, Feng-Ling Xu, Ling-Zhen Wang, Yi-Lin Wang, Ning-Ling Wang, Cheng-Zhu Liu, Xue-Dong Wu, Zhi-Biao Wang, Jiao Jin, Hong-Lan Yang

**Affiliations:** 1 Hematology and Oncology Follow-Up Center, Key Laboratory of Pediatric Hematology and Oncology of China, Ministry of Health, and National Children’s Medical Center (Shanghai), Shanghai Children’s Medical Center, Shanghai Jiao Tong University School of Medicine, Shanghai, China; 2 Department of Global Pediatric Medicine, St. Jude Children’s Research Hospital, Memphis, TN USA; 3 Department of Oncology, St. Jude Children’s Research Hospital, Memphis, TN USA; 4 Department of Pediatrics, Qilu Hospital of Shandong University, Jinan, China; 5 Department of Pediatrics and Adolescent Medicine, Hong Kong Children’s Hospital, The Chinese University of Hong Kong, Hong Kong, China; 6 Department of Oncology, Shanghai Children’s Medical Center, Shanghai Jiao Tong University School of Medicine, Shanghai, China; 7 Department of Hematology, Shanghai Children’s Medical Center, Shanghai Jiao Tong University School of Medicine, Shanghai, China; 8 Department of Paediatrics, Hospital for Sick Children, Toronto, Canada; 9 Department of Epidemiology and Cancer Control, St. Jude Children’s Research Hospital, Memphis, TN USA; 10 School of Pharmacy, The Chinese University of Hong Kong, Hong Kong, China; 11 Hong Kong Hub of Paediatric Excellence, The Chinese University of Hong Kong, Hong Kong, China

**Keywords:** Childhood cancer survivors, China, Long-term follow-up, Practice guidelines

## Abstract

**Background:**

Childhood cancer survivors (CCSs) are at increased risk of long-term treatment-related complications. Although international guidelines support risk-based long-term follow-up (LTFU) care, its standardized implementation in China has been limited. To address this gap, the National Children’s Medical Center–Shanghai convened a multidisciplinary expert panel to develop disease-based LTFU care plans tailored to the Chinese healthcare context.

**Methods:**

Guided by established international frameworks (Children’s Oncology Group, International Guideline Harmonization Group, and PanCareFollowUp), an expert group representing 25 institutions across China developed consensus-based LTFU care plans for common pediatric cancer patients and post-hematopoietic cell transplant survivors. Each care plan includes core components: a treatment summary, risk stratification for late effects, recommended surveillance, psychosocial evaluation, and lifestyle guidance. The panel also developed a consensus on the specific roles of oncologists, primary care providers, and subspecialists.

**Results:**

Finalized care plans provide structured, risk-adapted follow-up pathways for CCSs. The model emphasizes multidisciplinary collaboration, clinical feasibility, and scalability across diverse settings. As part of the care process, a centralized survivorship database has been integrated to facilitate clinical use and data collection. This system supports the generation of standardized treatment summaries and longitudinal documentation of late effects across the continuum of survivorship care. Tools, such as clinician checklists and survivor education templates, were also developed to support clinical use and promote consistency across institutions. A list of outcome metrics was proposed to evaluate the implementation outcomes of this initiative.

**Conclusions:**

This expert consensus establishes an innovative, nationally coordinated, disease-specific LTFU care framework for CCSs in China. This study provides a practical foundation for improving survivorship care quality and guiding clinical practice nationwide. This model can serve as a blueprint for other low- and middle-income countries seeking to strengthen LTFU care for CCSs.

**Graphical abstract:**

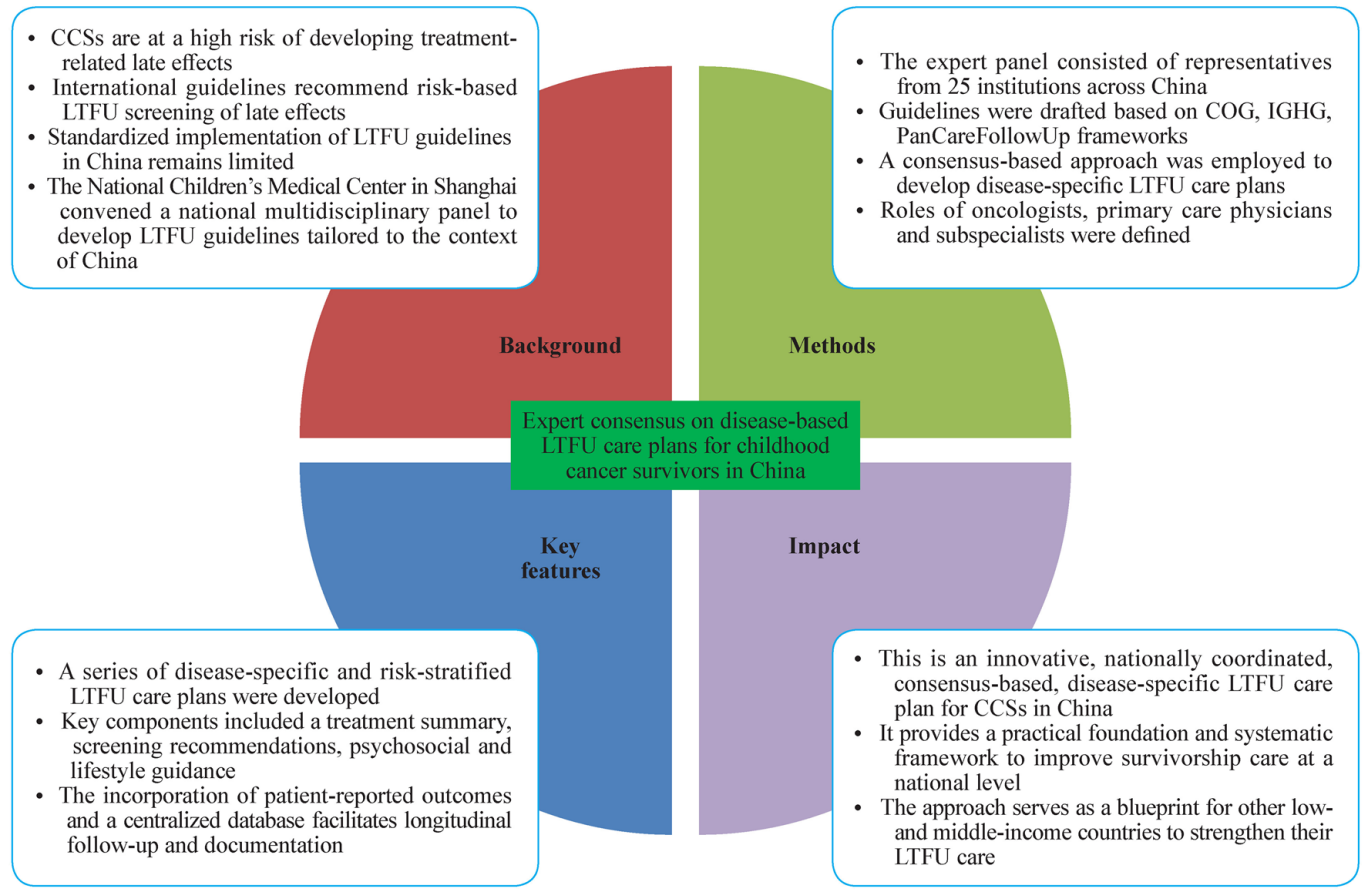

**Supplementary Information:**

The online version contains supplementary material available at 10.1007/s12519-025-00989-1.

## Introduction

Survivorship care for childhood cancer survivors (CCSs) has become an essential component of pediatric oncology practice, driven by significant advances in treatment that have markedly improved survival outcomes. Currently, over 80% of children diagnosed with cancer are expected to achieve long-term survival [1, 2]. However, these survivors remain at elevated risk for a spectrum of late effects, including secondary malignancies, cardiovascular disorders, endocrine dysfunctions, neurocognitive impairments, and psychosocial challenges [3–5]. Many of these complications may not manifest until years or even decades after the completion of therapy, highlighting the essential need for structured long-term follow-up (LTFU) care to facilitate early detection of late effects and opportunities for interventions to improve health-related quality of life [6].

Although survivorship guidelines have been developed by several international consortia—such as the Children’s Oncology Group (COG) [7, 8], PanCareFollowUp [9, 10], and the International Guideline Harmonization Group (IGHG) [11]—the delivery of LTFU care remains fragmented and inconsistent globally. A major contributing factor is the lack of clear, disease-specific care pathways during the transition from pediatric oncology centers to primary care settings [12]. Primary care providers (PCPs), such as community-based pediatricians or general practitioners who are expected to oversee survivorship care, often lack specialized training in the monitoring and management of treatment-related late effects. This knowledge gap may result in delayed diagnosis of complications, suboptimal adherence to screening protocols, and, ultimately, increased morbidity among CCSs [13]. Furthermore, the involvement of PCPs in the cancer care continuum may not be applicable in some countries, such as China and India [14]. Owing to the differences in healthcare systems across countries and settings, identifying an appropriate LTFU screening model and referral systems requires the consensus of various key stakeholders.

Several international pediatric oncology groups have recommended the distribution of a survivorship care plan (SCP) to support cancer patients after treatment by providing guidance on follow-up care, monitoring for potential late effects of treatment, and providing recommendations for lifestyle changes to promote overall well-being [15, 16]. Although one study conducted in Hong Kong, China, revealed that SCPs enhanced cancer-related knowledge in Chinese CCSs [17], the collective evidence of SCPs’ effectiveness in improving health outcomes in cancer survivors remains inconsistent [18, 19]. Randomized trials suggest that SCPs have a limited impact on adherence to surveillance protocols or early detection of late effects [20]. Warner et al. identified multiple barriers to SCP implementation, including insufficient time, lack of standardized templates, and inadequate provider training [21], all of which hinder consistent application in clinical settings.

Although survivorship care is often led by pediatric oncology teams in China, most pediatric oncologists receive limited training in LTFU care and late effects management [22]. Their primary focus remains on active treatment and relapse monitoring, with survivorship care historically considered outside the scope of routine oncology practice. As a result, many providers lack familiarity with risk-based surveillance strategies, standardized follow-up protocols, and the spectrum and severity of late-onset toxicities. This gap in experience contributes to inconsistent follow-up practices and hinders the development of structured survivorship programs across institutions [22, 23]. The challenge is further compounded by the absence of standardized, disease-tailored LTFU care plans. Variability in clinical practice among healthcare providers, coupled with limited infrastructure and resources for survivor monitoring, contributes to inconsistencies in care delivery [22, 24]. Poor coordination between oncology teams and PCPs or other subspecialists exacerbates communication barriers and leads to fragmented care transitions [25]. Furthermore, both CCSs and their families frequently lack awareness of the potential late effects of treatment. Inadequate patient education and engagement remain significant barriers to achieving optimal adherence to recommended surveillance [26].

To address these gaps, the National Children’s Medical Center–Shanghai (NCMCs) established the NCMCs-LTFU Study Group in December 2024—a multi-institutional collaboration aimed at standardizing pediatric cancer survivorship care in China. Through expert consensus informed by international frameworks and tailored to the local healthcare context, this report outlines the consensus methodology for developing a set of standardized disease-based LTFU care plans for common pediatric malignancies. It also describes an integrated electronic tool to support clinical implementation and a set of metrics to evaluate implementation outcomes. In contrast to SCPs, which refer to the general framework used in oncology to summarize treatment and outline follow-up, disease-based LTFU care plans are structured, with diagnosis- and exposure-specific tools designed to provide tailored surveillance and multidisciplinary guidance. The overarching goal of this work is to establish an LTFU model that emphasizes feasibility, multidisciplinary collaboration, and data-driven care in China.

## Methods

### Formation of the multidisciplinary expert panel

This expert consensus was developed by the NCMCs-LTFU Study Group, a national network of 25 pediatric oncology institutions in China, between October 2024 and July 2025. A multidisciplinary panel (*n* = 37) comprising pediatric hematologists (*n* = 2), pediatric oncologists (*n* = 25), survivorship specialists (*n* = 2), pediatric neurologists (*n* = 2), psychologists (*n* = 1), nurses (*n* = 3), and allied healthcare professionals (*n* = 2) was convened to draft disease-based LTFU care plans tailored to the Chinese healthcare context. These members served as key medical care providers and active onsite investigators of multicentered clinical trials in China and/or held leadership positions in their respective institutions or within the Chinese Children Cancer Group and/or were currently providing or planning to implement LTFU services at their institutions.

### Consensus development process

The development process drew upon established international survivorship guidelines, including those from the COG [7, 27, 28], IGHG [11, 29], and PanCareFollowUp [9, 10]. In defining treatment exposures, we aimed to be as comprehensive as possible, incorporating internationally recognized regimens (e.g., COG, International Society of Pediatric Oncology) as well as commonly utilized protocols in China. All relevant agents featured in contemporary treatment protocols were included to facilitate the identification of potential risks according to the actual treatments their patients received. Three local investigators from the core team (JC, YTC, and WYT) developed the first draft of the disease-based LTFU care plans in consultation with an international survivorship expert (MMH). The subsequent consensus process included multiple rounds of drafting, internal review, and external expert feedback, followed by iterative revision until agreement was reached within NCMCs-LTFU Study Group. Perspectives from patients/parents were incorporated indirectly via needs assessments and survivorship education feedback.

Between December 2024 and July 2025, the expert panel convened for three meetings (two onsite and one online; total 8 hours). A 3-hour onsite education session was held for representatives from 20 institutions in conjunction with the National Bone Marrow Transplantation Forum in Wuhan on July 19, 2025. Thirty minutes of the session were devoted to an overview of the evidence supporting the value of disease-specific care plans, followed by a 2-hour session on how to apply the Cancer Institute’s Common Terminology Criteria for Adverse Events (CTCAE) grading and the use of the centralized electronic Hematology and Oncology Long-Term Follow-Up System (HOLFS) database for documentation. The remaining 30 minutes were reserved for questions and discussions.

This work aimed to achieve consensus in three key components:

1. Development of disease-based LTFU care plans

The LTFU care plans were adapted to reflect local patterns of diagnosis and treatment, healthcare infrastructure, and follow-up practices. Each plan includes a structured treatment summary, individualized risk stratification, recommended surveillance, and delineated responsibilities across oncology, primary care, and subspecialty teams. Supporting materials—including care plan templates, clinician checklists, and educational tools for survivors and families—were also developed to support implementation. The expert panel also provided their perspectives on the specific responsibilities across oncology, primary care, and subspecialty disciplines to support coordinated care on the basis of existing guidelines.

To facilitate clinical use and promote consistency, each disease-specific care plan was designed to be compatible with the HOLFS—a centralized survivorship database developed by the NCMCs-LTFU Study Group. The electronic database was designed to document cancer history, treatment exposures, and long-term late effects experienced by CCSs. The expert panel provided suggestions on the essential features of this system on the basis of the literature [30–32], such as compliance with privacy regulations, user-friendly interfaces, robust data entry, data analytics capabilities to identify trends, and features to support the generation of standardized treatment summaries.

(2) Evaluation of medical and patient-reported outcomes

The expert panel reviewed a list of 31 relevant health conditions on the basis of the screening recommendations in the LTFU care plans, their practice experience, and a consensus from a previous study that focused on identifying high-priority late effects for harmonization of screening guidelines within China [23].

To capture a comprehensive view of survivors’ health-related quality of life and functional outcomes, the expert panel reviewed the relevant tools from the Chinese versions of the Patient-Reported Outcomes Measurement Information System (PROMIS) to select assessment tools for key domains, including global health, psychological distress, fatigue, physical function, social participation, and cognitive function. The choice of tools was based on their content validity and relevance to Chinese children and adolescent survivors.

(3) Implementation process and evaluation

It was decided a priori that the targeted eligible participants for implementation of this initiative would include CCSs who were diagnosed before the age of 18; who had completed curative-intent treatment—including chemotherapy, radiotherapy, surgery, and/or hematopoietic stem cell transplantation (HSCT)—at least two years prior to enrollment; and who were currently without evidence of active disease.

Key strategies to facilitate the implementation and evaluation of outcomes were discussed via the RE-AIM framework, which assesses interventions across five key domains: reach, effectiveness, adoption, implementation, and maintenance [33, 34]. The expert panel deliberated on and finalized the list of evaluation measures for each dimension.

This study was approved by the Ethics Committee of Shanghai Children’s Medical Center, Shanghai, China (approval number: SCMCIRB-K2025098). Given that this consensus work did not involve the collection of new patient-identifiable data, informed consent was waived.

## Results

### Structure of the disease-based LTFU care plans

**Fig. 1:**
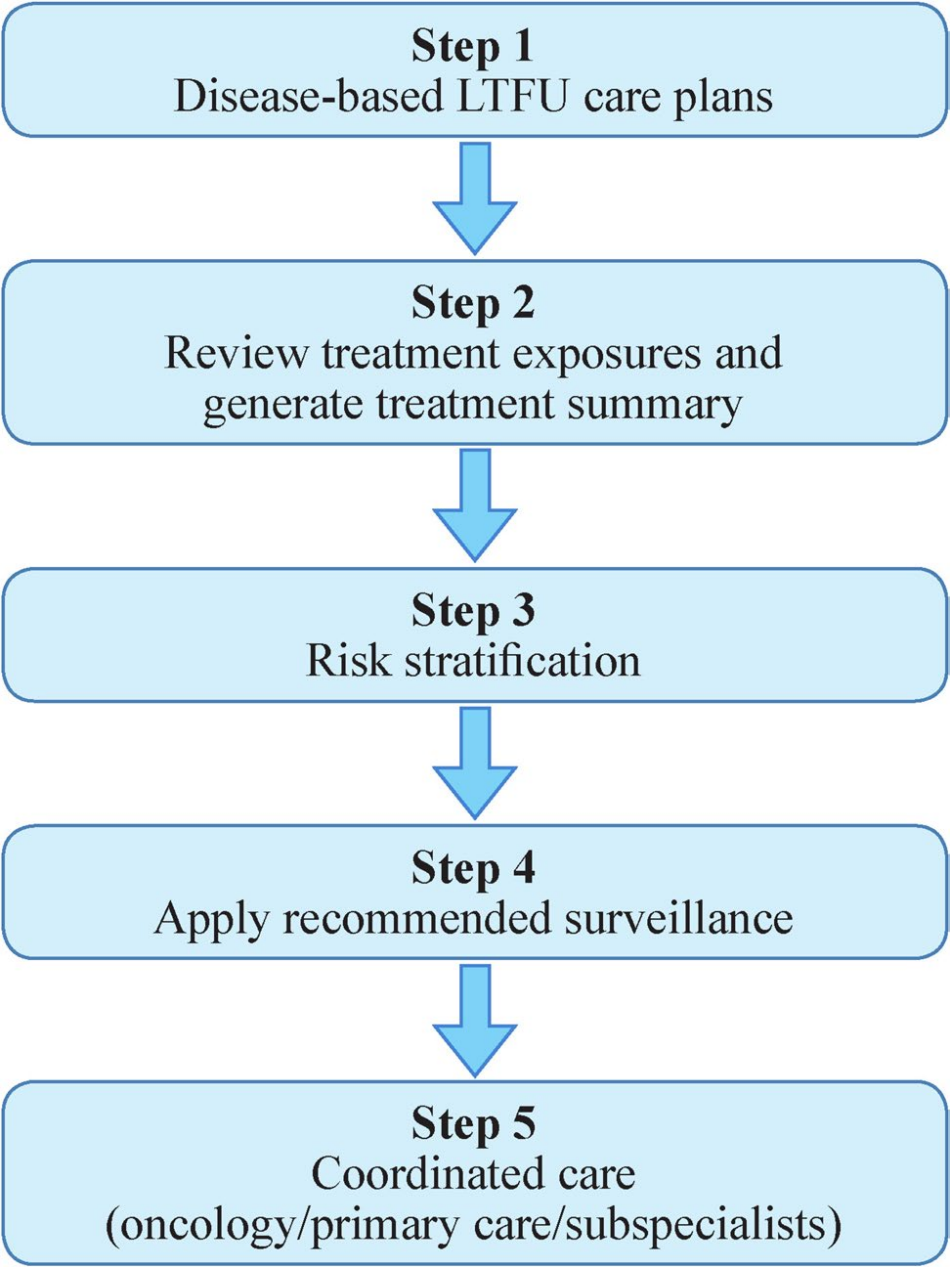
Flowchart showing the key steps in applying disease-based long-term follow-up (LTFU) care plans

The expert panel finalized standardized LTFU care plans for major pediatric malignancies, including acute lymphoblastic leukemia, hepatoblastoma, Hodgkin lymphoma, non-Hodgkin lymphoma, Wilms tumor, neuroblastoma, brain tumors, acute myeloid leukemia, and post-HSCT survivors. A simplified flowchart summarizing the key steps in applying disease-based LTFU care plans is presented in Fig. 1. A table summarizing disease-based LTFU care recommendations is provided to assist clinicians in quickly referencing key points. The comprehensive generic systems-based LTFU care plan is presented in Table 1, and the disease-specific LTFU care plan for childhood neuroblastoma patients is shown in Table 2. Additional disease-based LTFU care plans are provided in Supplementary Table 1. To enhance clarity and transparency, a simplified grading system was used, as shown in Table 1: strong consensus = supported by international guidelines and high expert agreement; moderate consensus = agreement with some limitations in evidence; and expert opinion = primarily expert panel judgment in areas with limited direct evidence. The consensus aims to support standardized practices and promote long-term health among survivors. Each plan follows a uniform structure comprising the following components.

**Table 1:** Comprehensive generic systems-based long-term follow-up care plan

System/organ	Therapeutic exposure	History/examination/consultation	Recommended screening	Strength of recommendation
General	Any chemotherapy	Perform physical examination: measure height and weight for body mass index calculation, and blood pressure Educate about potential late effects and purpose of LTFU Counsel on health protective behaviors Refer to nutrition/rehabilitation services Assess psychosocial functioning Provide educational/vocational support Refer for fertility consultation Recommend age-appropriate immunizations		Strong consensus
Cardiovascular	Anthracyclines (e.g., doxorubicin, daunorubicin) or chest/mediastinal irradiation	History: shortness of breath, dyspnea, chest pain, palpitations Physical: blood pressure, cardiac exam	Electrocardiograph and echocardiogram based on anthracycline dose and risk category. Cardiometabolic monitoring (fasting glucose, lipids)	Strong consensus
Endocrine/growth/delayed development	Cranial/spinal irradiation, TBI, abdominal irradiation high-dose alkylating agents, or HSCT	History: poor developmental progress, onset and tempo of puberty, hormonal use Physical: height, weight, body mass index and pubertal staging	Thyroid function (thyroid stimulating hormone, free T4), fasting glucose, lipids, luteinizing hormone and follicle-stimulating hormone, estradiol/testosterone	Moderate consensus
Hearing (ototoxicity)	Cisplatin, carboplatin, or cranial irradiation	History: hearing difficulties, tinnitus; vertigo Physical: otoscopic exam	Audiometry	Strong consensus
Visual impairment	Eye tumor or optic nerve involvement, orbital/cranial irradiation, busulfan, or corticosteroids	History: visual disturbances, blurry vision, visual neglect Physical: range of eye movement, nystagmus, ocular abnormalities	Ophthalmologic examination, screening for cataracts, retinopathy, and visual acuity decline	Moderate consensus
Neurocognitive	History of CNS tumor, cranial irradiation, high-dose methotrexate or cytarabine, intrathecal therapy, or HSCT	History: unable to focus, easily distracted, concerns on poorer memory/forgetfulness, learning difficulties, slow response	Formal neuropsychological assessment, CNS-vital sign and PROMIS cognitive function modules	Expert opinion
Renal/urinary	Ifosfamide, cisplatin, HSCT, nephrectomy, or abdominal irradiation	History: fatigue, changes in urine output or color, swelling Physical: blood pressure	Urinalysis, serum creatinine, blood urea nitrogen, electrolytes. Glomerular filtration rate estimation for high-risk exposures	Strong consensus
Pulmonary	Chest irradiation, bleomycin, busulfan, or HSCT	History: cough, wheezing, shortness of breath, dyspnea on exertion Physical: pulmonary exam	Pulmonary function test	Moderate consensus
Liver function	Liver irradiation, HSCT, chronic viral hepatitis	History: jaundice, ascites, spleen/liver enlargement Physical: abdominal exam	Alanine transaminase, aspartate transaminase, bilirubin	Strong consensus
Reproductive and sexual health	Sellar/suprasellar tumor; exposure to TBI, pelvic irradiation, alkylating agents, heavy metals, CNS irradiation, HSCT	History: onset and tempo of puberty; sexual functioning; menstrual history (girls); pregnancy history Physical: Tanner staging and growth until sexually mature	Luteinizing hormone and follicle-stimulating hormone, estradiol/testosterone. Endocrine referral if no signs of puberty by age 14 for boys/13 for girls	Moderate consensus
Bone health	Dexamethasone, prednisone, HSCT	History: atraumatic/fragility fractures	Dual-energy x-ray absorptiometry Bone health specialist referral if *Z*-score > 2 SD below the mean; if *Z*-score > 1 SD and < 2 SD below the mean, evaluation for hormonal deficiencies (e.g., growth hormone deficiency) and consultation with a bone health specialist	Moderate consensus
Mental health disorders	Any cancer experience or post-HSCT	History: social isolation/peer relationship difficulties/emotional/behavioral/mood disturbances	PROMIS-based patient-reported outcomes to monitor psychological functioning and life satisfaction Referral to social work, psychology, or psychiatry as clinically indicated	Moderate consensus
Secondary malignancies	Any radiation exposure, anthracycline, etoposide, alkylators, HSCT, germline predisposition	History: skin lesions, bleeding, easy bruising, changing moles, thyroid nodules, headaches, vomiting, cognitive/motor-sensory, deficits, seizures Physical: dermatologic exam, thyroid exam, neurologic exam, pulmonary exam, breast exam	Thyroid ultrasound as clinically indicated Breast: annual mammography/MRI from age 25 or 8 y post-radiation (whichever comes last) [28] Colorectal: every 5 y colonoscopy starting from age 30 or 5 y post-abdominal/pelvic irradiation (whichever comes last) [28]	Strong consensus

*LTFU* long-term follow-up, *TBI* total body irradiation, *HSCT* hematopoietic stem cell transplantation, *CNS* central nervous system, *PROMIS* Patient-Reported Outcomes Measurement Information System, *SD* standard deviations

**Table 2:** Disease-specific long-term follow-up care plan for childhood neuroblastoma

Health risk	Therapeutic exposures	History/examination/consultation (yearly)	Screening	Frequency
General	Any chemotherapy	Perform physical examination: measure height and weight for body mass index calculation, and blood pressure Educate about potential late effects and purpose of LTFU Counsel on health protective behaviors Refer to nutrition/rehabilitation services Assess psychosocial functioning Provide educational/vocational support Refer for fertility consultation Recommend age-appropriate immunizations		
Cardiac toxicity	Doxorubicin	History: shortness of breath, dyspnea, chest pain, palpitations Physical: blood pressure; cardiac exam	Electrocardiograph	Baseline at entry into LTFU, repeat as clinically indicated
			Echocardiogram	Every 2 y (doxorubicin equivalent dose > 250 mg/m^2^ or ≥ 100 mg/m^2^ to < 250 mg/m^2^ combined with chest irradiation ≥ 15 Gy or any doxorubicin combined with chest irradiation ≥ 30 Gy chest radiation [28] Every 5 y (doxorubicin equivalent dose ≥ 100 mg/m^2^ to < 250 mg/m^2^ combined with chest irradiation < 15 Gy or doxorubicin equivalent dose < 100 mg/m^2^ combined with chest irradiation 15 Gy to < 30 Gy [28]
Ototoxicity	Carboplatin, cisplatin	History: hearing difficulties, tinnitus, vertigo Physical: otoscopic exam	Pure-tone audiometry testing	Yearly, for patients ages ≤ 5 y and for all patients with established hearing loss Every 2 y, for patients ages 6–12 y; every 5 y for patients ages ≥ 13 y
Bladder toxicity	Cyclophosphamide	History: hematuria, urinary urgency/frequency, urinary incontinence/retention, dysuria, nocturia, abnormal urinary stream	Urinalysis	Yearly
Renal toxicity	Carboplatin, cisplatin	History: fatigue, changes in urine output or color, swelling	Blood pressure	Yearly
			Serum creatinine, blood urea nitrogen, electrolytes	Baseline at entry into LTFU, repeat as clinically indicated
Pulmonary toxicity	Irradiation potentially exposing the lungs	History: cough, wheezing, shortness of breath, dyspnea on exertion Physical: pulmonary exam	Pulmonary function tests	Baseline at entry into LTFU, repeat as clinically indicated and prior to general anesthesia
Liver function	Irradiation potentially exposing the liver	History: jaundice, ascites, spleen/liver enlargement Physical: abdominal exam	Alanine transaminase, aspartate transaminase, bilirubin	Baseline at entry into LTFU, repeat as clinically indicated
Delayed puberty/infertility	Carboplatin, cisplatin, cyclophosphamide, thiotepa, melphalan	History: onset and tempo of puberty, menstrual history (girls), sexual functioning, pregnancy history, hormonal use Physical: Tanner staging and growth until sexually mature	Luteinizing hormone and follicle-stimulating hormone, estradiol/testosterone	As clinically indicated
			Endocrine referral	If no signs of puberty by age 14 for boys/13 for girls
Impaired glucose metabolism/diabetes mellitus, dyslipidemia	Abdominal irradiation	Physical: height, weight, body mass index	Fasting blood glucose, hemoglobin A1c and lipids	Every 2 y, repeat as clinically indicated
Dental abnormalities	Any chemotherapy	Physical: oral exam, dental exam and cleaning	Evaluation by dentist	Every 6 mon
Musculoskeletal growth problems	Any irradiation	History: functional activity and limitations Physical: height, weight, sitting height		Yearly
Peripheral sensory or motor neuropathy	Carboplatin, cisplatin, vincristine	History: weakness, foot drop, paresthesias, dysesthesias Physical: neurologic exam	Neurologic exam	Yearly, until 3 y after therapy, monitor yearly if symptoms persist
			Physical therapy referral	For symptomatic neuropathy
Subsequent hematological malignancies	Heavy metals: carboplatin, cisplatin; cyclophosphamide, doxorubicin; thiotepa, melphalan	History: fatigue, bleeding, easy bruising; Physical: dermatologic exam (pallor, petechiae, purpura)	Complete blood count with differential and platelets	As clinically indicated
Subsequent solid neoplasms (skin, liver, lung, etc.)	Any irradiation	History: skin lesions, changing moles; welling/mass in soft tissues Physical: skin exam; abdominal exam; pulmonary exam	Imaging and other diagnostic tests	As clinically indicated

*LTFU* long-term follow-up

#### Treatment summary

The structured treatment summary includes the patient’s cancer diagnosis, age at diagnosis, treatment timeline, and detailed information on chemotherapy, radiotherapy, surgery, and HSCT or chimeric antigen receptor T-cell therapy exposure. Standardized data fields were developed for direct entry into the HOLFS, which can automatically generate a printable summary to support risk stratification and care continuity.

#### Shared care among a multidisciplinary team

Responsibilities across disciplines were explicitly delineated to support coordinated care: pediatric oncologists and survivorship providers oversee risk assessment and monitoring of late effects; PCPs support routine health maintenance and reinforce follow-up recommendations; and subspecialists manage specific complications, such as cardiomyopathy, endocrine disorders, or neurocognitive impairment.

#### Health promotion and preventive care

Plans include guidance on vaccination, nutrition, physical activity, smoking avoidance, and sun protection. Educational materials promote self-management and adherence.

#### Individualized evidence-based recommendations for organ-specific surveillance

These recommendations are stratified by treatment exposure, patient age, and risk level, and encompass three key domains—symptom inquiry, physical examination, and diagnostic testing.

### Evaluation of medical and patient-reported outcomes

Consistent with the literature and major pediatric oncology groups [3–5], late effects are defined as “health problems related to cancer treatment that persist or develop 2 or more years after completion of therapy”. To facilitate future research efforts, the expert panel agreed that the data collection for health conditions should follow standardized definitions and severity grading criteria for long-term and late-onset effects on the basis of the methods of the St. Jude Lifetime Cohort study. This approach represents a modified version of the National CTCAE, adapted to be more applicable to CCSs [35]. This consistent grading of health conditions would facilitate pooled analysis of surveillance data for future late effects surveillance statistics across centers.

The PROMIS Pediatric-48 Profile will be used to assess the various health-related quality-of-life dimensions in CCSs [36]. It is a collection of 7 and 8-item short forms assessing anxiety, depressive symptoms, fatigue, pain interference, physical function–mobility, and peer relationships, as well as a single pain-intensity item. The PROMIS Pediatric and Parent Proxy Short Form-Cognitive Function 7a will be used to assess cognitive functioning in everyday life, encompassing memory, attention, processing speed, and executive functions. The PROMIS tools have been validated in the Chinese pediatric population and have demonstrated reliability and validity in assessing health-related quality of life among children and adolescents in China [37, 38].

Consistent with the cultural norms of most Asian societies, the expert panel identified academic stress as an integral component of the experience of Chinese children and adolescents [39]. Academic stress in survivors who are still attending school will be assessed via the Education Stress Scale for Adolescents (ESSA). The 16-item ESSA addresses five latent variables: pressure related to studies, academic workload, concern about grades, self-expectation, and despondency [40]. The ESSA has been culturally adapted and validated in Chinese adolescents [40].

The HOLFS electronic database will be utilized to facilitate the documentation of cancer history, treatment exposures, and late effects experienced by CCSs. The expert panel identified the following components as high-priority features of the electronic database: (1) standardized data entry; (2) longitudinal tracking of late effects; and (3) automated generation of treatment summaries—thereby supporting risk-based care delivery and multidisciplinary coordination. The prototype of the HOLFS was further optimized in April 2025 to integrate the above features and was piloted for use at three institutions in Shanghai, Shenzhen, and Hong Kong.

### Implementation process and evaluation

Starting in August 2025, disease-based LTFU care plans will be introduced at selected institutions via structured implementation strategies, including (1) clinical provider training through educational workshops and handbooks; (2) the integration of care plans into the HOLFS and clinical workflows, facilitating coordinated care and reducing administrative burdens; and (3) the dissemination of caregiver- and survivor-facing materials for risk-based education. This will include tailored survivorship education materials, digital health tools, and self-management support to improve adherence to follow-up recommendations and ensure timely interventions. Patient partners and survivors from non-governmental organizations, such as CureKids China [41], will be engaged to provide feedback on education materials and self-management interventions.

The project-specific evaluation measures for each dimension based on the RE-AIM framework are listed in the following section, and the project-specific evaluation measures for each dimension are detailed in Table 3:

**Table 3:** Measures, data sources, and data collection timeline for disease-based long-term follow-up care plans

Assessment level	Measures	Data sources	Timeline
Reach	Individual level: number and characteristics of patients enrolled, patient recruitment rate, demographic comparison with total eligible population	Patient registries, medical records, patient surveys	Ongoing
	Organizational level: ability of healthcare systems to identify eligible patients, presence of patient tracking systems, referral processes	Clinic records, administrative databases, provider interviews	Baseline, midpoint, post-implementation
Effectiveness	Primary outcomes: disease progression, adherence to medications and treatment regimens, hospitalizations, emergency visits	Electronic health records, patient follow-up data	Baseline, annual review
	Secondary outcomes: patient-reported quality of life, symptom management, functional status, satisfaction with care	Standardized patient-reported outcome measures (e.g., PROMIS), patient interviews	Baseline, annual review
Adoption	Organizational level: number and type of providers adopting LTFU care plans, institutional policies supporting LTFU care	Clinic policies, provider surveys, hospital/clinic administrative data	Baseline, post-implementation
	Perceptions of healthcare providers regarding the usability of LTFU plans	Provider interviews, focus groups	Midpoint, post-implementation
Implementation	Individual level: patient adherence to scheduled follow-ups, medication adherence, use of self-management tools	EHR, medication refill data, patient logs	Ongoing, annual review
	Organizational level: provider adherence to LTFU protocols, staff training completion, use of decision-support tools	Training logs, implementation reports, provider feedback	Baseline, midpoint, post-implementation
	Cost analysis: staffing requirements, resource utilization	Budget records, healthcare cost data	Post-implementation
Maintenance	Individual level: individual level: adherence to LTFU, sustained health improvements, ongoing symptom control	Follow-up patient surveys, medical records review	12 mon, long-term (2+ y)
	Organizational level: integration of LTFU care into routine healthcare practice, continued financial and policy support. Clinic policies, stakeholder interviews, business plans	Clinic policies, stakeholder interviews, business plans	Post-implementation, long-term assessment

*LTFU* long-term follow-up, *PROMIS* patient-reported outcomes measurement information system

#### Reach measurement

The enrollment rates of eligible survivors across sites will be analyzed for equitable access, with identification through HOLFS records and oncology team referrals. Educational sessions for families promote awareness and participation, and their feedback is taken for evaluation.

#### Effectiveness assessment

Outcomes will include adherence to follow-up, detection of late effects, and PROs, measured through standardized PRO surveys on quality of life.

#### Adoption focus

This metric will evaluate how well the LTFU team integrates care plans into clinical workflows, measured by engaging oncology, primary care, and survivorship teams while identifying barriers through feedback surveys.

#### Implementation evaluation

The fidelity and feasibility of care plan delivery will be assessed via structured workflows among healthcare providers and supportive tools to improve care coordination and patient engagement. Some examples of indicators of success are the development of protocols tailored to the institution and the completion and adherence rates of care plans**.**

#### Maintenance

Long-term adherence to care plans, ongoing training, and quality improvement will be evaluated, along with the potential for institutionalization and expansion of care plans to other centers after the project concludes.

## Discussion

This expert consensus represents the first nationally coordinated effort in China to establish structured, disease-specific LTFU care plans for CCSs across 25 institutions in the country. By incorporating international guidelines and adapting them to the local healthcare context, this initiative addresses longstanding gaps in post-treatment survivorship care across pediatric oncology institutions. Importantly, this consensus lays the groundwork for a national survivorship strategy tailored to the structure and resource availability of the Chinese healthcare system. The lessons learned from the practical implementation of the disease-specific LTFU care plan aim to inform future policy, provider-training frameworks, and digital health integration for long-term survivorship care. Moreover, this consensus provides a foundational model for improving survivorship care in China and may inform similar efforts in other low- and middle-income countries seeking to build national frameworks for CCS follow-up.

In contrast to general SCPs, these disease-specific protocols offer tailored, risk-based guidance that improves the relevance and applicability of follow-up strategies. The integration of HOLFS provides a practical and scalable platform for implementation, enables automated treatment summary generation, structured documentation, and multidisciplinary coordination, and applies standardized definitions and grading criteria for long-term and late-onset effects on the basis of the methods of the St. Jude Lifetime Cohort study [35]. To facilitate implementation, the HOLFS database was initially introduced in 20 hospitals within the NCMCs-LTFU study group through structured training, with progressive expansion planned to additional centers. For providers who do not yet have access to HOLFS, care plans can also be applied through manual completion of the treatment summary templates [9], which may be completed manually. This approach ensures that care plans align with evidence-based recommendations while also considering the unique resource limitations and healthcare settings in China. We also emphasize that the development of this consensus and standardized care plans does not imply that every institution must implement these guidelines. Each institution should have the autonomy to modify or adapt the guidelines according to its specific timeline and context. However, at a broader level, we hope that this initiative will contribute to optimizing the implementation of future guidelines on the national front in the foreseeable future. To ensure sustainability, care plans are updated in alignment with international guideline revisions, generally every five years or sooner if major changes occur.

Importantly, the inclusion of PROMIS-based PROs and detailed organ-specific surveillance recommendations reflects a holistic approach to survivorship that addresses both clinical and psychosocial needs. Delineating responsibilities across oncology, primary care, and subspecialty teams fosters shared accountability and improves care continuity. In the important context of achieving the goals outlined in Healthy China 2030 [42], the country has been actively introducing and evaluating new reforms to the Chinese healthcare delivery system. The use of PROs for holistic and patient-centered care and the establishment of referral networks to promote a shared-care approach are in line with national efforts, ensuring that tens of thousands of CCSs can receive appropriate follow-up care and support, facilitating their reintegration into daily life and helping them achieve optimal quality of life in the coming decades.

Despite its strengths, this consensus is not without limitations. Although the panel lacked direct representation from endocrinology, surgery, neurosurgery, and radiotherapy, their perspectives were considered both prior to and during the guideline development process. We plan a broader inclusion of various specialties in future updates and intervention recommendations. Its recommendations were based primarily on expert review and adaptation of existing international frameworks; prospective validation across diverse institutions and survivor populations is still needed. Furthermore, integrating care into community-based settings remains a challenge, given the limited survivorship experience among non-oncology providers. Finally, we acknowledge that China is a geographically large region and that differences in resources and constraints must inevitably exist across institutions in the country. However, the initiation of this national endeavor toward a standardized set of care plans represents the first step in optimizing the collaborative process of survivorship guidelines and intervention development within China. Future work will focus on evaluating the effectiveness and sustainability of LTFU care in pediatric oncology, as the initiative is rolled out at various institutions within the NCMCs-LTFU Study Group.

In conclusion, this expert consensus marks a significant milestone in establishing structured, disease-specific LTFU care plans for CCSs in China. The adaptation of international guidelines to the local healthcare context addresses critical gaps in survivorship care and lays the foundation for a national survivorship strategy. The integration of PROs, promotion of shared care among multidisciplinary teams, and systematic documentation of survivorship outcomes aims to enhance the retention of survivors in LTFU programs and promote lifelong holistic care. While there are limitations, such as the need for prospective validation and challenges in integrating care into various settings, this consensus represents a vital first step toward improving pediatric cancer survivorship care in China. The model can also serve as a blueprint for other low- and middle-income countries seeking to strengthen follow-up care for CCSs.

## Supplementary Information

Below is the link to the electronic supplementary material.

Supplementary file 1 (PDF 201 KB)

## Data Availability

Data sharing is not applicable to this article as no data sets were generated or analyzed during the current study.

## References

[1] Ehrhardt MJ, Krull KR, Bhakta N, Liu Q, Yasui Y, Robison LL, et al. Improving quality and quantity of life for childhood cancer survivors globally in the twenty-first century. Nat Rev Clin Oncol. 2023;20:678–96. 37488230 10.1038/s41571-023-00802-w

[2] Dixon SB, Liu Q, Chow EJ, Oeffinger KC, Nathan PC, Howell RM, et al. Specific causes of excess late mortality and association with modifiable risk factors among survivors of childhood cancer: a report from the Childhood Cancer Survivor Study cohort. Lancet. 2023;401:1447–57. 37030315 10.1016/S0140-6736(22)02471-0 PMC10149583

[3] Armstrong GT, Kawashima T, Leisenring W, Stratton K, Stovall M, Hudson MM, et al. Aging and risk of severe, disabling, life-threatening, and fatal events in the childhood cancer survivor study. J Clin Oncol. 2014;32:1218–27. 24638000 10.1200/JCO.2013.51.1055 PMC3986385

[4] Bhakta N, Liu Q, Ness KK, Baassiri M, Eissa H, Yeo F, et al. The cumulative burden of surviving childhood cancer: an initial report from the St Jude Lifetime Cohort Study (SJLIFE). Lancet. 2017;390:2569–82. 28890157 10.1016/S0140-6736(17)31610-0 PMC5798235

[5] Suh E, Stratton KL, Leisenring WM, Nathan PC, Ford JS, Freyer DR, et al. Late mortality and chronic health conditions in long-term survivors of early-adolescent and young adult cancers: a retrospective cohort analysis from the Childhood Cancer Survivor Study. Lancet Oncol. 2020;21:421–35. 32066543 10.1016/S1470-2045(19)30800-9 PMC7392388

[6] Armstrong GT, Liu Q, Yasui Y, Neglia JP, Leisenring W, Robison LL, et al. Late mortality among 5-year survivors of childhood cancer: a summary from the childhood cancer survivor study. J Clin Oncol. 2009;27:2328–38. 19332714 10.1200/JCO.2008.21.1425 PMC2677921

[7] Landier W, Bhatia S, Eshelman DA, Forte KJ, Sweeney T, Hester AL, et al. Development of risk-based guidelines for pediatric cancer survivors: the children’s oncology group long-term follow-up guidelines from the children’s oncology group late effects committee and nursing discipline. J Clin Oncol. 2004;22:4979–90. 15576413 10.1200/JCO.2004.11.032

[8] DeVine A, Landier W, Hudson MM, Constine LS, Bhatia S, Armenian SH, et al. The Children’s Oncology Group long-term follow-up guidelines for survivors of childhood, adolescent, and young adult cancers: a review. JAMA Oncol. 2025;11:544–53. 39976936 10.1001/jamaoncol.2024.6812 PMC12188901

[9] van Kalsbeek RJ, Mulder RL, Haupt R, Muraca M, Hjorth L, Follin C, et al. The pancarefollowup care intervention: a European harmonised approach to person-centred guideline-based survivorship care after childhood, adolescent and young adult cancer. Eur J Cancer. 2022;162:34–44. 34953441 10.1016/j.ejca.2021.10.035

[10] Michel G, Mulder RL, van der Pal HJH, Skinner R, Bárdi E, Brown MC, et al. Evidence-based recommendations for the organization of long-term follow-up care for childhood and adolescent cancer survivors: a report from the PanCareSurFup guidelines working group. J Cancer Surviv. 2019;13:759–72. 31396878 10.1007/s11764-019-00795-5

[11] Kremer LC, Mulder RL, Oeffinger KC, Bhatia S, Landier W, Levitt G, et al. A worldwide collaboration to harmonize guidelines for the long-term follow-up of childhood and young adult cancer survivors: a report from the International Late Effects of Childhood Cancer Guideline Harmonization Group. Pediatr Blood Cancer. 2013;60:543–9. 23281199 10.1002/pbc.24445 PMC3819170

[12] Ehrhardt MJ, Friedman DN, Hudson MM. Health care transitions among adolescents and young adults with cancer. J Clin Oncol. 2024;42:743–54. 38194608 10.1200/JCO.23.01504 PMC11264196

[13] McCabe MS, Partridge AH, Grunfeld E, Hudson MM. Risk-based health care, the cancer survivor, the oncologist, and the primary care physician. Semin Oncol. 2013;40:804–12. 24331199 10.1053/j.seminoncol.2013.09.004 PMC4465133

[14] Cai J, Cheung YT, Hudson MM. Care models and barriers to long-term follow-up care among childhood cancer survivors and health care providers in Asia: a literature review. JCO Glob Oncol. 2024;10:e2300331. 38452303 10.1200/GO.23.00331 PMC10939639

[15] Dixon SB, Bjornard KL, Alberts NM, Armstrong GT, Brinkman TM, Chemaitilly W, et al. Factors influencing risk-based care of the childhood cancer survivor in the 21st century. CA Cancer J Clin. 2018;68:133–52. 29377070 10.3322/caac.21445 PMC8893118

[16] Dixon SB, Chow EJ, Hjorth L, Hudson MM, Kremer LCM, Morton LM, et al. The future of childhood cancer survivorship: challenges and opportunities for continued progress. Pediatr Clin North Am. 2020;67:1237–51. 33131544 10.1016/j.pcl.2020.07.013 PMC7773506

[17] Ma CT, Chou HW, Lam TTN, Tung YT, Lai YW, Lee LK, et al. Provision of a personalized survivorship care plan and its impact on cancer-related health literacy among childhood cancer survivors in Hong Kong. Pediatr Blood Cancer. 2023;70:e30084. 36383479 10.1002/pbc.30084

[18] Birken SA, Urquhart R, Munoz-Plaza C, Zizzi AR, Haines E, Stover A, et al. Survivorship care plans: are randomized controlled trials assessing outcomes that are relevant to stakeholders? J Cancer Surviv. 2018;12:495–508. 29572602 10.1007/s11764-018-0688-6 PMC6054562

[19] Hill RE, Wakefield CE, Cohn RJ, Fardell JE, Brierley ME, Kothe E, et al. Survivorship care plans in cancer: a meta-analysis and systematic review of care plan outcomes. Oncologist. 2020;25:e351–72. 32043786 10.1634/theoncologist.2019-0184 PMC7011634

[20] Kadan-Lottick NS, Ross WL, Mitchell HR, Rotatori J, Gross CP, Ma X. Randomized trial of the impact of empowering childhood cancer survivors with survivorship care plans. J Natl Cancer Inst. 2018;110:1352–9. 29771337 10.1093/jnci/djy057

[21] Warner EL, Wu YP, Hacking CC, Wright J, Spraker-Perlman HL, Gardner E, et al. An assessment to inform pediatric cancer provider development and delivery of survivor care plans. J Cancer Educ. 2015;30:677–84. 25893925 10.1007/s13187-015-0829-9

[22] Cai J, Malone S, Bhakta N, Pui CH, Chen J, Hu S, et al. Accessibility of and barriers to long-term follow-up care for childhood cancer survivors. JAMA Netw Open. 2024;7:e2440258. 39418017 10.1001/jamanetworkopen.2024.40258 PMC11581527

[23] Cheung YT, Zhang H, Cai J, Au-Doung LWP, Yang LS, Yan C, et al. Identifying priorities for harmonizing guidelines for the long-term surveillance of childhood cancer survivors in the Chinese Children Cancer Group (CCCG). JCO Glob Oncol. 2021;7:261–76. 33591820 10.1200/GO.20.00534 PMC8081494

[24] Lee JW, Yeo Y, Ju HY, Cho HW, Yoo KH, Sung KW, et al. Current status and physicians’ perspectives of childhood cancer survivorship in Korea: a nationwide survey of pediatric hematologists/oncologists. J Korean Med Sci. 2023;38:e230. 37489718 10.3346/jkms.2023.38.e230 PMC10366409

[25] Breij D, Hjorth L, Bouwman E, Walraven I, Kepak T, Kepakova K, et al. Healthcare providers’ expected barriers and facilitators to the implementation of person-centered long-term follow-up care for childhood cancer survivors: a PanCareFollowUp study. Cancer Med. 2024;13:e70225. 39440690 10.1002/cam4.70225 PMC11497108

[26] Knighting K, Kirton JA, Thorp N, Hayden J, Appleton L, Bray L. A study of childhood cancer survivors” engagement with long-term follow-up care: “to attend or not to attend, that is the question”. Eur J Oncol Nurs. 2020;45:101728. 32163861 10.1016/j.ejon.2020.101728

[27] Kong AP, Choi KC, Li AM, Hui SS, Chan MH, Wing YK, et al. Association between physical activity and cardiovascular risk in Chinese youth independent of age and pubertal stage. BMC Public Health. 2010;10:303. 20525239 10.1186/1471-2458-10-303 PMC2893096

[28] Children’s Oncology Group. Long-term follow-up guidelines for survivors of childhood, adolescent and young adult cancers. Version 6.0. 2023. http://www.survivorshipguidelines.org/. Accessed 22 Jul 2025. 10.1001/jamaoncol.2024.6812 PMC12188901 39976936

[29] Ehrhardt MJ, Leerink JM, Mulder RL, Mavinkurve-Groothuis A, Kok W, Nohria A, et al. Systematic review and updated recommendations for cardiomyopathy surveillance for survivors of childhood, adolescent, and young adult cancer from the International Late Effects of Childhood Cancer Guideline Harmonization Group. Lancet Oncol. 2023;24:e108–20. 37052966 10.1016/S1470-2045(23)00012-8

[30] Poplack DG, Fordis M, Landier W, Bhatia S, Hudson MM, Horowitz ME. Childhood cancer survivor care: development of the Passport for Care. Nat Rev Clin Oncol. 2014;11:740. 25348788 10.1038/nrclinonc.2014.175 PMC5142740

[31] Warrington L, Absolom K, Conner M, Kellar I, Clayton B, Ayres M, et al. Electronic systems for patients to report and manage side effects of cancer treatment: systematic review. J Med Internet Res. 2019;21:e10875. 30679145 10.2196/10875 PMC6365878

[32] Shliakhtsitsava K, Eary R, Hughes A, Betts AC, Cochran C, Eshelman-Kent D, et al. The development of a dynamic electronic health records database for childhood cancer survivors at a single institution: 30 years of data for clinical and research applications. Pediatr Blood Cancer. 2025;72:e31882. 40629666 10.1002/pbc.31882

[33] Glasgow RE, Vogt TM, Boles SM. Evaluating the public health impact of health promotion interventions: the RE-AIM framework. Am J Public Health. 1999;89:1322–7. 10474547 10.2105/ajph.89.9.1322 PMC1508772

[34] Glasgow RE, Harden SM, Gaglio B, Rabin B, Smith ML, Porter GC, et al. RE-AIM planning and evaluation framework: adapting to new science and practice with a 20-year review. Front Public Health. 2019;7:64. 30984733 10.3389/fpubh.2019.00064 PMC6450067

[35] Hudson MM, Ehrhardt MJ, Bhakta N, Baassiri M, Eissa H, Chemaitilly W, et al. Approach for classification and severity grading of long-term and late-onset health events among childhood cancer survivors in the St. Jude lifetime cohort. Cancer Epidemiol Biomarkers Prev. 2017;26:666–74. 28035022 10.1158/1055-9965.EPI-16-0812 PMC5413397

[36] HealthMeasures. Available PROMIS^®^ measures for pediatric self-report (ages 8–17) and available PROMIS^®^ measures for early childhood parent-report (ages 1–5) and parent proxy (ages 5–17). 2025. https://www.healthmeasures.net/explore-measurement-systems/promis/intro-to-promis/list-of-pediatric-measures. Accessed 22 Jul 2025.

[37] Chan SWW, Chien CW, Wong AYL, Pang MYC. Translation and psychometric validation of the traditional Chinese version of patient-reported outcomes measurement information system Pediatric-25 Profile version 2.0 (PROMIS-25) in Chinese children with cancer in Hong Kong. Qual Life Res. 2021;30:1779–91. 33770335 10.1007/s11136-021-02759-8

[38] Li D, Zong X, Huang Q, Wu F, Huang Y, Ge Y, et al. Validation of the simplified Chinese version of PROMIS parent proxy-25 profile in parents of children with cancer. J Pediatr Nurs. 2023;72:e19–26. 37331836 10.1016/j.pedn.2023.05.016

[39] Dunne M, Sun J, Nguyen D, Thai TT, Loan K, Dixon J. The influence of educational pressure on the mental health of adolescents in East Asia: methods and tools for research. J Sci. 2010. https://api.semanticscholar.org/CorpusID:54888252. Accessed 22 Jul 2025.

[40] Sun J, Dunne MP, Hou XY, Xu AQ. Educational stress scale for adolescents. J Psychoeduc Assess. 2011;29:534–46.

[41] CureKids China. 2025. https://www.curekids.cn/. Accessed 22 Jul 2025.

[42] World Health Organization. Healthy China 2030 (from vision to action). 2025. https://www.who.int/teams/health-promotion/enhanced-wellbeing/ninth-global-conference/healthy-china. Accessed 22 Jul 2025.

