# Supplementary material for: Expert consensus on disease-based long-term follow-up care plans for childhood cancer survivors
Source: World J Pediatr. 2025 Oct 30;21(12):1204–17. doi: 10.1007/s12519-025-00989-1 (PMC12678504; doi:10.1007/s12519-025-00989-1)
Supplement: Supplementary file 1 — (PDF 201 KB) [file 12519_2025_989_MOESM1_ESM.pdf]

Supplementary Table 1. Additional disease-based LTFU care plans for acute lymphoblastic leukemia (**a**), Hodgkin lymphoma (**b**), non-Hodgkin lymphoma (**c**), hepatoblastoma (**d**), Wilms tumor (**e**), acute myeloid leukemia or post-hematopoietic stem cell transplantation (**f**), brain tumors (**g**)

a. Late-effects screening recommendation for childhood acute lymphoblastic leukemia

Health risk	Therapeutic exposures	History/ examination/ consultation	Screening	Frequency
**General**	Any chemotherapy	Perform physical examination: measure height and weight for body mass index calculation, and blood pressure<br>Educate about potential late effects and purpose of LTFU<br>Counsel on health protective behaviors<br>Refer to nutrition/rehabilitation services prn<br>Assess psychosocial functioning<br>Provide educational/vocational support prn<br>Refer for fertility consultation as requested<br>Recommend age-appropriate immunizations		
**Cardiac toxicity**	Daunorubicin	History: shortness of breath, dyspnea, chest pain, palpitations<br>Physical: blood pressure; cardiac exam	Electrocardiograph	Baseline at entry into LTFU, repeat as clinically indicated
			Echocardiogram	No screening with doxorubicin equivalent dose < 100 mg/m^2
**Bladder toxicity**	Cyclophosphamide	History: hematuria, urinary urgency/ frequency, urinary incontinence/retention, dysuria nocturia, abnormal urinary stream	Urinalysis	Yearly
**Liver function**	Methotrexate, high dose methotrexate, mercaptopurine	History: jaundice, ascites, spleen/liver enlargement<br>Physical: abdominal exam	ALT, AST, bilirubin	Baseline at entry into LTFU, repeat as clinically indicated

**Delayed puberty/ infertility**	Cyclophosphamid, TBI, HSCT	History: onset and tempo of puberty<br>Physical: Tanner staging and growth until sexually mature	Estradiol, testosterone, AMH, LH/FSH	As clinically indicated
			Endocrine referral	If no signs of puberty by age 14 for boys/13 for girls
**Thyroid dysfunction**	TKI, TBI	History: fatigue, cold intolerance, constipation, depressed mood<br>Physical: thyroid exam	TSH, free T4	Yearly
**Metabolic syndrome**	TBI	Physical: height, weight, body mass index	Fasting blood glucose, hemoglobin A1c and lipids	Every 2 y
**Dental abnormalities**	Any chemotherapy	Physical: oral exam, dental exam and cleaning	Evaluation by dentist	Every 6 mon
**Cataracts**	Dexamethasone, prednisone, TBI	History: visual changes<br>Physical: visual acuity, funduscopic exam	Evaluation by ophthalmologist or optometrist	Yearly
**Bone mineral density deficit**	Dexamethasone, prednisone	History: atraumatic/fragility fractures	DXA	Baseline at entry into LTFU, repeat as clinically indicated
			Bone health specialist referral	If *Z*-score > 2 SD below the mean; if *Z*-score > 1 and < 2 SD below the mean, evaluation for hormonal deficiencies (e.g., GH deficiency) and consultation with a bone health specialist
**Osteonecrosis**	Dexamethasone,	History: joint pain	MRI	As clinically indicated

	prednisone	Physical: musculoskeletal exam		
**Peripheral sensory or motor neuropathy**	Vincristine	History: weakness, foot drop, paresthesias, dysesthesias<br>Physical: neurologic exam	Neurologic exam	Yearly, until 2 to 3 y after therapy, monitor yearly if symptoms persist
			Physical therapy referral	For symptomatic neuropathy
**Neurocognitive deficits**	HD/IT methotrexate, cytarabine, CNS irradiation	History: educational and/or vocational progress	Neuropsychological evaluation	Baseline at entry into LTFU, repeat as clinically indicated
**Hypogammaglobulinemia**	HSCT with any history of chronic GVHD	History: recurrent unusual infections	Quantitative immunoglobulins	Baseline at entry into LTFU, repeat as clinically indicated
**Subsequent hematological malignancies**	Daunorubicin; cyclophosphamide	History: fatigue, bleeding, easy bruising<br>Physical: dermatologic exam (pallor, petechiae, purpura)	CBC with differential and platelets	As clinically indicated
**Subsequent solid neoplasms (skin, thyroid, CNS, etc.)**	HSCT, TBI	History: skin lesions, changing moles, thyroid nodules, headaches, vomiting, cognitive/motor-sensory, deficits, seizures<br>Physical: skin exam, thyroid exam, neurologic exam	Imaging and other diagnostic tests	As clinically indicated

LTFU long-term follow-up, *ALT* alanine aminotransferase, *AST* aspartate aminotransferase, *TBI* total body irradiation, *HSCT* hematopoietic stem cell transplantation, *AMH* anti-Mullerian hormone, *LH* luteinizing hormone, *FSH* follicle-stimulating hormone, *TKI* tyrosine kinase inhibitor, *TSH* thyroid stimulating hormone, *DXA* dual-energy x-ray absorptiometry, *GH* growth hormone, *IT* intrathecal therapy, *CNS* central nervous system, *GVHD* chronic graft versus host disease, *CBC* complete blood count, *MRI* magnetic resonance imaging, *SD* standard deviation

b. Late-effects screening recommendation for childhood Hodgkin lymphoma

**Health risk**	**Therapeutic exposures**	**History/ examination/ consultation (yearly)**	**Screening**	**Frequency**
**General**	Any chemotherapy	Perform physical examination: measure height and weight for body mass index calculation, and blood pressure<br>Educate about potential late effects and purpose of LTFU<br>Counsel on health protective behaviors<br>Refer to nutrition/rehabilitation services prn<br>Assess psychosocial functioning<br>Provide educational/vocational support prn<br>Refer for fertility consultation as requested<br>Recommend age-appropriate immunizations		
**Cardiac toxicity**	Doxorubicin	History: shortness of breath, dyspnea, chest pain, palpitations<br>Physical: blood pressure; cardiac exam	Electrocardiograph	Baseline at entry into LTFU, repeat as clinically indicated
			Echocardiogram	Every 2 y (doxorubicin equivalent dose > 250 mg/m^2 or ≥ 100 mg/m^2 to < 250 mg/m^2 combined with chest irradiation ≥15 Gy or any doxorubicin combined with chest irradiation ≥ 30 Gy<br>Every 5 y (doxorubicin equivalent dose ≥ 100 mg/m^2 to < 250 mg/m^2 combined with chest irradiation < 15 Gy or doxorubicin equivalent dose <

				100 mg/m^2 combined with chest irradiation 15 Gy to < 30 Gy
**Bladder toxicity**	Cyclophosphamide	History: hematuria, urinary urgency/ frequency, urinary incontinence/retention, dysuria, nocturia, abnormal urinary stream	Urinalysis	Yearly
**Pulmonary toxicity**	Bleomycin, irradiation potentially exposing the lungs	History: cough, wheezing, shortness of breath, dyspnea on exertion<br>Physical: pulmonary exam	Pulmonary function tests	Baseline at entry into LTFU, repeat as clinically indicated and prior to general anesthesia
**Liver function**	Radiation potentially exposing the liver	History: jaundice, ascites, spleen/liver enlargement<br>Physical: abdominal exam	ALT, AST, bilirubin	Baseline at entry into LTFU, repeat as clinically indicated
**Delayed puberty/ infertility**	Cyclophosphamide	History: onset and tempo of puberty, menstrual history (girls), sexual functioning, pregnancy history, hormonal use<br>Physical: Tanner staging and growth until sexually mature	Estradiol, testosterone, AMH, LH/FSH	As clinically indicated
			Endocrine referral	If no signs of puberty by age 14 for boys/13 for girls
**Thyroid dysfunction**	Irradiation potentially exposing the thyroid gland	History: fatigue, cold intolerance, constipation, depressed mood<br>Physical: thyroid exam	TSH, free T4-	Yearly
**Impaired glucose metabolism/**	Abdominal irradiation	Physical: height, weight, body mass index	Fasting blood glucose, hemoglobin A1c	Every 2 y, repeat as clinically indicated

**diabetes mellitus, dyslipidemia**			and lipids	
**Dental abnormalities**	Any chemotherapy	Physical: oral exam, dental exam and cleaning	Evaluation by dentist	Every 6 mon
**Cataracts**	Prednisone	History: visual changes<br>Physical: visual acuity, funduscopic exam	Evaluation by ophthalmologist or optometrist	Yearly
**Bone mineral density deficit**	Prednisone	History: atraumatic/fragility fractures	DXA	Baseline at entry into LTFU, repeat as clinically indicated
			Bone health specialist referral	If *Z*-score > 2 SD below the mean; if *Z*-score > 1 and < 2 SD below the mean, evaluation for hormonal deficiencies (e.g., GH deficiency) and consultation with a bone health specialist
**Osteonecrosis**	Prednisone	History: joint pain<br>Physical: musculoskeletal exam	MRI as clinically indicated	
**Musculoskeletal growth problems**	Any radiation	History: functional activity and limitations<br>Physical: height, weight, sitting height		Yearly
**Peripheral sensory or motor neuropathy**	Vincristine, vinblastine	History: weakness, foot drop, paresthesias, dysesthesias<br>Physical: neurologic exam	Neurologic exam	Yearly, until 3 y after therapy, monitor yearly if symptoms persist
			Physical therapy referral	For symptomatic neuropathy
**Subsequent hematological**	Doxorubicin, cyclophospham	History: fatigue, bleeding, easy	CBC with differential and	As clinically indicated

**malignancies**	ide	bruising<br>Physical: dermatologic exam (pallor, petechiae, purpura)	platelets	
**Subsequent solid neoplasms (skin, thyroid, breast, colorectal, etc.)**	Any irradiation	History: skin lesions, changing moles; swelling/mass in soft tissues<br>Physical: skin exam; abdominal exam; pulmonary exam	Imaging and other diagnostic tests	As clinically indicated<br>For any breast irradiation: yearly beginning at age 25 and 8 y after irradiation<br>For any abdomen/pelvis irradiation: every 5 y, beginning at age 30 and 5 y after irradiation

LTFU long-term follow-up, *ALT* alanine aminotransferase, *AST* aspartate aminotransferase, *AMH* anti-Mullerian hormone, *LH* luteinizing hormone, *FSH* follicle-stimulating hormone, *TSH* thyroid stimulating hormone, *DXA* dual-energy x-ray absorptiometry, *GH* growth hormone, *CBC* complete blood count, *MRI* magnetic resonance imaging, *SD* standard deviation

c. Late-effects screening recommendation for childhood non-Hodgkin lymphoma

Health risk	Therapeutic exposures	History/ examination/ consultation (yearly)	Screening	Frequency
**General**	Any chemotherapy	Perform physical examination: measure height and weight for body mass index calculation, and blood pressure<br>Educate about potential late effects and purpose of LTFU<br>Counsel on health protective behaviors<br>Refer to nutrition/rehabilitation services prn<br>Assess psychosocial functioning<br>Provide educational /vocational support prn<br>Refer for fertility consultation as requested<br>Recommend age-appropriate immunizations		
**Cardiac toxicity**	Doxorubicin	History: shortness of breath, dyspnea, chest pain, palpitations<br>Physical: blood pressure; cardiac	Electrocardiograph	Baseline at entry into LTFU, repeat as clinically indicated
			Echocardiogram	Every 2 y (doxorubicin equivalent dose > 250 mg/m^2 or ≥ 100

		exam		mg/m^2 to < 250 mg/m^2 combined with chest irradiation ≥15 Gy or any doxorubicin combined with chest irradiation ≥ 30 Gy<br>Every 5 y (doxorubicin equivalent dose ≥ 100 mg/m^2 to < 250 mg/m^2 combined with chest irradiation < 15 Gy or doxorubicin equivalent dose < 100 mg/m^2 combined with chest irradiation 15 Gy to < 30 Gy
**Bladder toxicity**	Cyclophosphamide	History: hematuria, urinary urgency/ frequency, urinary incontinence/retention, dysuria, nocturia, abnormal urinary stream	Urinalysis	Yearly
**Liver function**	Methotrexate	History: jaundice, ascites, spleen/liver enlargement<br>Physical: abdominal exam	ALT, AST, bilirubin	Baseline at entry into LTFU, repeat as clinically indicated
**Delayed puberty/ Infertility**	Cyclophosphamide	History: onset and tempo of puberty, menstrual history (girls), sexual functioning, pregnancy history, hormonal use<br>Physical: Tanner staging and growth until sexually mature	Estradiol, testosterone, AMH, LH/FSH	As clinically indicated
			Endocrine referral	If no signs of puberty by age 14 for boys/13 for girls

**Thyroid dysfunction**	TKI	History: fatigue, cold intolerance, constipation, depressed mood<br>Physical: thyroid exam	TSH, free T4	Yearly
**Dental abnormalities**	Any chemotherapy	Physical: oral exam, dental exam and cleaning	Evaluation by dentist	Every 6 mon
**Cataracts**	Prednisone	History: visual changes<br>Physical: visual acuity, funduscopic exam	Evaluation by ophthalmologist or optometrist	Yearly
**Bone mineral density deficit**	Prednisone	History: atraumatic/fragility fractures	DXA	Baseline at entry into LTFU, repeat as clinically indicated
			Bone health specialist referral	If *Z*-score > 2 SD below the mean; if *Z*-score > 1 and < 2 SD below the mean, evaluation for hormonal deficiencies (e.g., GH deficiency) and consultation with a bone health specialist
**Osteonecrosis**	Prednisone	History: joint pain<br>Physical: musculoskeletal exam	MRI as clinically indicated	
**Peripheral sensory or motor neuropathy**	Vincristine	History: weakness, foot drop, paresthesias, dysesthesias<br>Physical: neurologic exam	Neurologic exam	Yearly, until 3 y after therapy, monitor yearly if symptoms persist
			Physical therapy referral	For symptomatic neuropathy
**Neurocognitive deficits**	HD/IT methotrexate	History: educational and/or vocational progress	Neuropsychological evaluation	Baseline at entry into LTFU, repeat as clinically indicated

**Hypogammaglobulinemia**	Rituximab	History: recurrent unusual infections	Quantitative immunoglobulins	Baseline at entry into LTFU, repeat as clinically indicated
**Subsequent hematological malignancies**	Doxorubicin, cyclophosphamide	History: fatigue, bleeding, easy bruising<br>Physical: dermatologic exam (pallor, petechiae, purpura)	CBC with differential and platelets	As clinically indicated

LTFU long-term follow-up, *ALT* alanine aminotransferase, *AST* aspartate aminotransferase, *AMH* anti-Mullerian hormone, *LH* luteinizing hormone, *FSH* follicle-stimulating hormone, *TKI* tyrosine kinase inhibitor, *TSH* thyroid stimulating hormone, *DXA* dual-energy x-ray absorptiometry, *GH* growth hormone, *IT* intrathecal therapy, *CBC* complete blood count, *MRI* magnetic resonance imaging, *SD* standard deviation

d. Late-effects screening recommendation for childhood hepatoblastoma[a]

Health risk	Therapeutic exposures	History/ examination/ consultation (yearly)	Screening	Frequency
**General**	Any chemotherapy	Perform physical examination: measure height and weight for body mass index calculation, and blood pressure<br>Educate about potential late effects and purpose of LTFU<br>Counsel on health protective behaviors<br>Refer to nutrition/rehabilitation services prn<br>Assess psychosocial functioning<br>Provide educational/vocational support prn<br>Refer for fertility consultation as requested<br>Recommend age-appropriate immunizations		
**Cardiac toxicity**	Doxorubicin	History: shortness of breath, dyspnea, chest pain, palpitations<br>Physical: blood pressure, cardiac exam	Electrocardiograph	Baseline at entry into LTFU, repeat as clinically indicated
			Echocardiogram	Every 2 y (doxorubicin equivalent dose > 250 mg/m^2)
**Ototoxicity**	Carboplatin, cisplatin	History: hearing difficulties, tinnitus, vertigo<br>Physical: otoscopic exam	Pure tone audiometry testing	Yearly, for patients ages ≤ 5 y and for all patients with established hearing loss<br>Every 2 y, for patients

				ages 6-12 y<br>Every 5 y for patients ages ≥ 13 y
**Renal toxicity**	Carboplatin, cisplatin	History: fatigue, changes in urine output or color, swelling	Blood pressure	Yearly
			BUN, creatinine, electrolytes	Baseline at entry into LTFU, repeat as clinically indicated
**Liver function**	5-Fluorouracil, hepatectom, liver irradiation	History: jaundice, ascites, spleen/liver enlargement<br>Physical: abdominal exam	ALT, AST, bilirubin	Baseline at entry into LTFU, repeat as clinically indicated
**Delayed puberty/ infertility**	Carboplatin, cisplatin	History: onset and tempo of puberty, menstrual history (girls), sexual functioning, pregnancy history, hormonal use<br>Physical: Tanner staging and growth until sexually mature	Estradiol, Testosterone, AMH, LH/FSH	As clinically indicated
			Endocrine referral	If no signs of puberty by age 14 for boys/13 for girls
**Impaired glucose metabolism/ diabetes mellitus, dyslipidemia**	Abdominal irradiation	Physical: height, weight, body mass index	Fasting blood glucose, hemoglobin A1c and lipids	Every 2 y, repeat as clinically indicated
**Pulmonary toxicity**	Irradiation potentially exposing the lungs	History: cough, wheezing, shortness of breath, dyspnea on exertion<br>Physical: pulmonary exam	Pulmonary function tests	Baseline at entry into LTFU, repeat as clinically indicated and prior to general anesthesia
**Dental abnormalities**	Any chemotherapy	Physical: oral exam, dental exam and cleaning	Evaluation by dentist	Every 6 mon
**Musculoskeletal growth problems**	Any irradiation	History: functional activity and limitations<br>Physical: height, weight, sitting		Yearly

		height		
**Peripheral sensory or motor neuropathy**	Carboplatin, cisplatin; vincristine	History: weakness, foot drop, paresthesias, dysesthesias<br>Physical: neurologic exam	Neurologic exam	Yearly, until 3 y after therapy, monitor yearly if symptoms persist
			Physical therapy referral	For symptomatic neuropathy
**Subsequent hematological malignancies**	Carboplatin, cisplatin; cyclophosph amide; doxorubicin	History: fatigue, bleeding, easy bruising<br>Physical: dermatologic exam (pallor, petechiae, purpura)	CBC with differential and platelets	As clinically indicated
**Subsequent solid neoplasms (skin, liver, lung, etc.)**	Any irradiation	History: skin lesions, changing moles, welling/mass in soft tissues<br>Physical: skin exam, abdominal exam, pulmonary exam	Imaging and other diagnostic tests	As clinically indicated

LTFU long-term follow-up, *BUN* blood urea nitrogen, *ALT* alanine aminotransferase, *AST* aspartate aminotransferase, *AMH* anti-Mullerian hormone, *LH* luteinizing hormone, *FSH* follicle-stimulating hormone, *CBC* complete blood count. [a]Late effects after liver transplant not included

e. Late-effects screening recommendation for childhood Wilms tumor

**Health risk**	**Therapeutic exposures**	**History/ examination/ consultation (yearly)**	**Screening**	**Frequency**
**General**	Any chemotherapy	Perform physical examination: measure height and weight for body mass index calculation, and blood pressure<br>Educate about potential late effects and purpose of LTFU<br>Counsel on health protective behaviors<br>Refer to nutrition/rehabilitation services prn<br>Assess psychosocial functioning<br>Provide educational/vocational support prn<br>Refer for fertility consultation as requested<br>Recommend age-appropriate immunizations		
**Cardiac toxicity**	Doxorubicin	History: shortness of breath, dyspnea, chest	Electrocardiograph	Baseline at entry into LTFU, repeat as clinically indicated

		pain, palpitations;<br>Physical: blood pressure; cardiac exam	Echocardiogram	Every 2 y (doxorubicin equivalent dose > 250 mg/m^2 or ≥ 100 mg/m^2 to < 250 mg/m^2 combined with chest irradiation ≥15 Gy or any doxorubicin combined with chest irradiation ≥ 30 Gy<br>Every 5 y (doxorubicin equivalent dose ≥ 100 mg/m^2 to < 250 mg/m^2 combined with chest irradiation < 15 Gy or doxorubicin equivalent dose < 100 mg/m^2 combined with chest irradiation 15 Gy to < 30 Gy
**Ototoxicity**	Carboplatin	History: hearing difficulties, tinnitus, vertigo<br>Physical: otoscopic exam	Pure tone audiometry testing	Yearly, for patients ages ≤ 5 y and for all patients with established hearing loss<br>Every 2 y, for patients ages 6-12 y; every 5 y for patients ages ≥ 13 y
**Bladder toxicity**	Cyclophosphamide	History: hematuria, urinary urgency/ frequency, urinary incontinence/retention, dysuria, nocturia, abnormal urinary stream	Urinalysis	Yearly
**Renal toxicity**	Carboplatin, nephrectomy	History: fatigue, changes in urine output or color, swelling	Blood pressure	Yearly
			BUN, creatinine, electrolytes	Baseline at entry into LTFU, repeat as clinically indicated
**Pulmonary**	Dactinomycin,	History: cough,	Pulmonary function	Baseline at entry into

**toxicity**	pulmonary irradiation	wheezing, shortness of breath, dyspnea on exertion<br>Physical: pulmonary exam	tests	LTFU, repeat as clinically indicated and prior to general anesthesia
**Liver function**	Irradiation potentially exposing liver	History: jaundice, ascites, spleen/liver enlargement<br>Physical: abdominal exam	ALT, AST, bilirubin	Baseline at entry into LTFU, repeat as clinically indicated
**Delayed puberty/ Infertility**	Carboplatin, cyclophosphamide	History: onset and tempo of puberty, menstrual history (girls), sexual functioning, pregnancy history, hormonal use<br>Physical: Tanner staging and growth until sexually mature	Estradiol, testosterone, AMH, LH/FSH	As clinically indicated
			Endocrine referral	If no signs of puberty by age 14 for boys/13 for girls
**Impaired glucose metabolism/ diabetes mellitus, dyslipidemia**	Abdominal irradiation	Physical: height, weight, body mass index	Fasting blood glucose, hemoglobin A1c and lipids	Every 2 y, repeat as clinically indicated
**Dental abnormalities**	Any chemotherapy	Physical: oral exam, dental exam and cleaning	Evaluation by dentist	Every 6 mon
**Musculoskeletal growth problems**	Any irradiation	History: functional activity and limitations<br>Physical: height, weight, sitting		Yearly

		height		
**Peripheral sensory or motor neuropathy**	Carboplatin, vincristine	History: weakness, foot drop, paresthesias, dysesthesias<br>Physical: neurologic exam	Neurologic exam	Yearly, until 3 y after therapy, monitor yearly if symptoms persist
			Physical therapy referral	For symptomatic neuropathy
**Subsequent hematological malignancies**	Carboplatin, cyclophosphamide, doxorubicin	History: fatigue, bleeding, easy bruising<br>Physical: dermatologic exam (pallor, petechiae, purpura)	CBC with differential and platelets	As clinically indicated
**Subsequent solid neoplasms (skin, liver, lung, etc.)**	Any irradiation	History: skin lesions, changing moles, welling/mass in soft tissues<br>Physical: skin exam, abdominal exam, pulmonary exam	Imaging and other diagnostic tests	As clinically indicated

LTFU long-term follow-up, *BUN* blood urea nitrogen, *ALT* alanine aminotransferase, *AST* aspartate aminotransferase, *AMH* anti-Mullerian hormone, *LH* luteinizing hormone, *FSH* follicle-stimulating hormone, *CBC* complete blood count

f. Late-effects screening recommendation for childhood acute myeloid leukemia or post-hematopoietic stem cell transplantation

Health risk	Therapeutic exposures	History/ examination/ consultation	Screening	Frequency
**General**	Any chemotherapy	Perform physical examination: measure height and weight for body mass index calculation, and blood pressure<br>Educate about potential late effects and purpose of LTFU<br>Counsel on health protective behaviors<br>Refer to nutrition/rehabilitation services prn		

		Assess psychosocial functioning<br>Provide educational/vocational support prn<br>Refer for fertility consultation as requested<br>Recommend age-appropriate immunizations		
**Cardiac toxicity**	Daunorubicin	History: shortness of breath, dyspnea, chest pain, palpitations<br>Physical: blood pressure; cardiac exam	Electrocardiograph	Baseline at entry into LTFU, repeat as clinically indicated
			Echocardiogram	Every 2 y (doxorubicin equivalent dose > 250 mg/m^2)
**Bladder toxicity**	TBI, HSCT	History: hematuria, urinary urgency/ frequency, urinary incontinence/retention, dysuria nocturia, abnormal urinary stream	Urinalysis	Yearly
**Renal toxicity**	TBI, HSCT	History: fatigue, changes in urine output or color, swelling	Blood pressure	Yearly
			BUN, creatinine, electrolytes	Baseline at entry into LTFU, repeat as clinically indicated
**Pulmonary toxicity**	TBI, HSCT, chronic GVHD	History: cough, wheezing, shortness of breath, dyspnea on exertion<br>Physical: pulmonary exam	Pulmonary function tests	Baseline at entry into LTFU, repeat as clinically indicated and prior to general anesthesia
**Liver function**	HSCT, chronic GVHD	History: Jaundice, ascites, spleen/liver enlargement<br>Physical: abdominal exam	ALT, AST, bilirubin	Baseline at entry into LTFU, repeat as clinically indicated
**Delayed puberty/ infertility**	TBI, HSCT	History: onset and tempo of puberty<br>Physical: Tanner staging and growth until sexually mature	Estradiol, testosterone, AMH, LH/FSH	As clinically indicated
			Endocrine referral	If no signs of puberty by age 14 for boys/13 for girls
**Thyroid**	TKI, TBI	History: fatigue,	TSH, free T4	Yearly

**dysfunction**		cold intolerance, constipation, depressed mood<br>Physical: thyroid exam		
**Metabolic syndrome**	TBI, HSCT	Physical: height, weight, body mass index	Fasting blood glucose, hemoglobin A1c and lipids	Every 2 y
**Dental abnormalities**	Any chemotherapy	Physical: oral exam, dental exam and cleaning	Evaluation by dentist	Every 6 mon
**Ocular toxicity**	HSCT with any history of chronic GVHD, TBI	History: visual changes<br>Physical: visual acuity, funduscopic exam	Evaluation by ophthalmologist or optometrist	Yearly
**Bone mineral density deficit**	HSCT	History: atraumatic/fragility fractures	DXA	Baseline at entry into LTFU, repeat as clinically indicated
			Bone health specialist referral	If *Z*-score > 2 SD below the mean; If *Z*-score > 1 and < 2 SD below the mean, evaluation for hormonal deficiencies (e.g., GH deficiency) and consultation with a bone health specialist
**Osteonecrosis**	HSCT	History: joint pain<br>Physical: musculoskeletal exam	MRI	As clinically indicated
**Neurocognitive deficits**	IT; cytarabine; CNS radiation	History: educational and/or vocational progress	Neuropsychological evaluation	Baseline at entry into LTFU, repeat as clinically indicated
**Dermatologic toxicity**	HSCT with chronic GVHD	History: vitiligo, sclerodermatous changes, alopecia, nail underdevelopment/ hypoplasia	Clinical skin exam; hair exam; nail exam	Yearly

		Physical: clinical skin exam, hair exam, nail exam		
**Immunologic complications**	HSCT with any history of chronic GVHD	History: chronic conjunctivitis, chronic sinusitis, chronic bronchitis, recurrent or unusual infections, sepsis	Quantitative immunoglobulins and lymphocyte subset analysis	Baseline at entry into LTFU, repeat as clinically indicated
**Subsequent hematological malignancies**	Daunorubicin; etoposide	History: fatigue, bleeding, easy bruising<br>Physical: dermatologic exam (pallor, petechiae, purpura)	CBC with differential and platelets	As clinically indicated
**Subsequent solid neoplasms (skin, thyroid, lung CNS, colorectal, breast, etc.)**	HSCT, TBI	History: skin lesions, changing moles, thyroid nodules, headaches, vomiting, cognitive/motor-sensory, deficits, seizures<br>Physical: skin exam, thyroid exam, neurologic exam, pulmonary exam, breast exam	Imaging and other diagnostic tests	As clinically indicated
			Colonoscopy	Every 5 y, beginning at age 30 y and 5 y after radiation (whichever comes last)
			Mammogram and breast MRI	Yearly, at age 25 y or 8 y after radiation, whichever comes last

LTFU long-term follow-up, *BUN* blood urea nitrogen, *ALT* alanine aminotransferase, *AST* aspartate aminotransferase, *TBI* total body irradiation, *HSCT* hematopoietic stem cell transplantation, *AMH* anti-Mullerian hormone, *LH* luteinizing hormone, *FSH* follicle-stimulating hormone, *TKI* tyrosine kinase inhibitor, *GH* growth hormone, *IT* intrathecal therapy, *CNS* central nervous system, *GVHD* chronic graft versus host disease, *CBC* complete blood count, *MRI* magnetic resonance imaging, *SD* standard deviation

g. Late-effects screening recommendation for childhood brain tumor survivors (medulloblastoma/CNS embryonal tumors/CNS germ cell tumors/ low-grade gliomas)

**Health risk**	**Therapeutic exposures**	**History/ examination/ consultation (yearly)**	**Screening**	**Frequency**
**General**	Any chemotherapy	Perform physical examination: measure height and weight for body mass index calculation, and blood pressure<br>Educate about potential late effects and purpose of LTFU<br>Counsel on health protective behaviors<br>Inquire about sleep disturbances<br>Encourage biannual dental check-up<br>Refer for nutrition/rehabilitative services prn<br>Assess psychosocial functioning<br>Provide educational/vocational support as needed<br>Refer for fertility consultation as requested<br>Recommend age-appropriate immunizations		
**Developmental delay/ neurocognitive deficits**	All	History: poor developmental progress, unable to focus, easily distracted, concerns on poorer memory/forgetfulness, learning difficulties, slow response	For < 6 y, comprehensive developmental assessment<br>For ≥ 6 y, neuropsychological assessment including attention monitoring	Baseline developmental/ neuropsychological assessment prior to cranial irradiation then repeat full assessment 1 y post irradiation<br>Consider repeat assessment if symptoms of cognitive decline/developmental regression or if clinically indicated<br>Yearly to every 2 y follow-up with history taking and physical examination<br>Follow-up at key stages, e.g., entering primary school/secondary school/prior to taking public exams, etc.
**Mental health disorders**	All	History: social isolation/peer relationship difficulties/emotional/behavioral and mood disturbances	Psychosocial screening with attention to: Social skills, depression, anxiety, post-traumatic stress, suicidal	Yearly

			ideation	
**Ototoxicity**	Carboplatin, cisplatin, cranial irradiation, patients with language delay/dysarticulation	History: hearing difficulties, tinnitus, vertigo<br>Physical: otoscopic exam	Pure tone audiometry testing	Yearly, for patients ages ≤ 5 y and for all patients with established hearing loss<br>Every 2 y, for patients ages 6-12 y; every 5 y for patients ages ≥ 13 y
**Visual impairment**	Brain tumor along the optic tract/occipital lobe; cranial irradiation, other relevant history of visual impairment	History: visual disturbances, blurry vision, visual neglect<br>Physical: range of eye movement, nystagmus, ocular abnormalities	Ophthalmological consultation including visual field and visual acuity	Baseline at entry into LTFU, repeat as clinically indicated
**Cardiac toxicity**	History of CSI irradiation	History: shortness of breath, dyspnea, chest pain, palpitations<br>Physical: blood pressure, cardiac exam	Electrocardiograph	Baseline at entry into LTFU, repeat as clinically indicated
			Echocardiogram	Every 2 y (doxorubicin equivalent dose > 250 mg/m^2 or ≥ 100 mg/m^2 to < 250 mg/m^2 combined with chest radiation ≥15 Gy or any doxorubicin combined with chest radiation ≥ 30 Gy chest radiation<br>Every 5 y (doxorubicin equivalent dose ≥ 100 mg/m^2 to < 250 mg/m^2 combined with chest radiation < 15 Gy or doxorubicin equivalent dose < 100 mg/m^2 combined with chest radiation 15 Gy to < 30 Gy
**Bladder toxicity**	Cyclophosphamide, Ifosfamide	History: hematuria, urinary urgency/frequency, urinary	Urinalysis	Yearly

		incontinence/retention, dysuria, nocturia, abnormal urinary stream		
**Renal toxicity**	Carboplatin, cisplatin, ifosfamide, bevacizumab or TKI	History: fatigue, changes in urine output or color, swelling, weakness, cramps	Blood pressure	Yearly
			BUN, creatinine, electrolytes; urine protein	Baseline at entry into LTFU, repeat as clinically indicated
**Pulmonary toxicity**	CSI	History: cough, wheezing, Shortness of breath, Dyspnea on exertion<br>Physical: pulmonary exam	Pulmonary function tests	Baseline at entry into LTFU, repeat as clinically indicated and prior to general anesthesia
**Liver function**	CSI	History: jaundice, ascites, spleen/liver enlargement<br>Physical: abdominal exam	ALT, AST, bilirubin	Baseline at entry into LTFU, repeat as clinically indicated
**Delayed puberty/ infertility**	Alkylating agents (CCNU, cyclophosphamide, ifosfamide, thiotepa), carboplatin, cisplatin, CNS irradiation, sellar/suprasellar tumors	History: onset and tempo of puberty, menstrual history (girls), sexual functioning, pregnancy history, hormonal use<br>Physical: Tanner staging and growth until sexually mature	Estradiol, testosterone, AMH, LH/FSH	As clinically indicated
			Endocrine referral	If no signs of puberty by age 14 for boys/13 for girls
**Endocrine problems**	All	Physical: height, weight, body mass index	Fasting blood glucose, hemoglobin A1c and lipids morning cortisol, thyroid function test (if given cranial irradiation)	Every 2 y, repeat as clinically indicated
**Musculoskeletal growth problems**	Any irradiation	History: functional activity and limitations<br>Physical: height, weight, sitting height		Yearly
**Peripheral**	Vinca alkaloid	History: weakness, foot	Neurologic exam	Yearly, until 3 y after

**sensory or motor neuropathy**	(vincristine, vinblastine), carboplatin, cisplatin, taxols	drop, paresthesia, dysesthesias<br>Physical: neurologic exam		therapy, monitor yearly and refer to see neurologist if symptoms persist
			Physical therapy referral	For symptomatic neuropathy
			Occupational therapy referral	Patients with foot drop for orthosis
**Subsequent hematological malignancies**	Etoposide, cyclophosphamide, thiotepam, melphalan	History: fatigue, bleeding, easy bruising<br>Physical: dermatologic exam (pallor, petechiae, purpura)	CBC with differential and platelets	As clinically indicated
**Subsequent solid neoplasms (CNS, skin, thyroid etc.)**	Any irradiation, germline predisposition	History: neurological symptoms, increased cranial pressure symptoms, skin lesions, changing moles, welling/mass in soft tissues, neck mass<br>Physical: neuro exam, skin exam, abdominal exam, pulmonary exam	Imaging and other diagnostic tests	As clinically indicated

CNS central nervous system, *LTFU* long-term follow-up, *BUN* blood urea nitrogen, *CSI* craniospinal irradiation, *TKI* tyrosine kinase inhibitor, *ALT* alanine aminotransferase, *AST* aspartate aminotransferase, *AMH* anti-Mullerian hormone, *LH* luteinizing hormone, *FSH* follicle-stimulating hormone, *CBC* complete blood count
